# Assessment of Risk Factors for Falls among Patients with Parkinson's Disease

**DOI:** 10.1155/2021/5531331

**Published:** 2021-09-28

**Authors:** Jacek Wilczyński, Magdalena Ścipniak, Kacper Ścipniak, Kamil Margiel, Igor Wilczyński, Rafał Zieliński, Piotr Sobolewski

**Affiliations:** Laboratory of Posturology, Collegium Medicum, Jan Kochanowski University in Kielce, Poland

## Abstract

**Introduction:**

The aim of this study was to assess the risk factors for falls in patients with Parkinson's disease.

**Materials and Methods:**

The study comprised 53 participants (52.8% women and 47.2% men). The Hoehn and Yahr 5-point disability scale was used to assess the severity of Parkinson's disease. The Tinetti Balance and Gait Scale were used to evaluate the risk of falls. The Katz scale was used to test the independence of people with PD. The Falls Efficacy Scale-International Short Form (FES-I) was implemented to assess fear of falling.

**Results:**

The majority of participants was at a high risk of falls, being at the same level for women and men. A significant relationship was noted between the risk of falls and subjective assessment of mobility (*χ*^2^ = 31.86, *p* < 0.001), number of falls (*χ*^2^ = 37.92, *p* < 0.001), independence of the subjects (*χ*^2^ = 19.28, *p* < 0.001), type of injury suffered during the fall (*χ*^2^ = 36.93, *p* < 0.001), external factors (*χ*^2^ = 33.36, *p* < 0.001), and the level of fear of falling (*χ*^2^ = 8.88, *p* < 0.001). A significant relationship also occurred between the number of falls and the fear of falling (*χ*^2^ = 33.49, *p* < 0.001) and between the number of falls and disease severity (*χ*^2^ = 45.34, *p* < 0.001). The applied physiotherapy did not reduce the risk of falls (*χ*^2^ = 3.18, *p* = 0.17).

**Conclusions:**

Individuals who rated their mobility as good or excellent were at a low risk of falls. People who fell more times were at a high risk of falling. People more independent were at a low risk of falls. Previous injuries were the most associated with being at risk of falling. Uneven surfaces and obstacles on one's path are the external factors most associated with the risk of falling. People with low levels of fall anxiety were at a low risk of falls. Most people with low fall anxiety have never fallen. Additionally, the majority of patients with stage 1 of the disease have not fallen at all. The reason for the ineffectiveness of physiotherapy may be due to the exercise programs used and the lack of systematic implementation of them. PD is different for each patient; thus, it is important to select individually customized physiotherapy depending on motor and nonmotor symptoms, as well as general health of a patient.

## 1. Introduction

In people with Parkinson's disease, falls occur more frequently in the general elderly population [1]. They are reported as experienced by 38-68%, constituting one of the most significant causes of morbidity and mortality [2, 3]. Recurrent falls often cause traumatic consequences and shorten the duration of life in patients with PD by 7 years [4, 5]. Clinical diagnosis of Parkinson's disease (PD) is based on the detection of at least 2 of its 3 symptoms: resting tremor, bradykinesia, and rigidity [6, 7]. Gradually, muscle stiffness and slowing down movement appear, as well as incorrect posture and pathological gait patterns [8, 9]. Other consequences of this disease are disturbances in postural stability and body balance [10, 11]. Due to increased muscle tension and stiffness, it is difficult to perform activities of daily living and freely move around—as a result of limitations in daily activity, people with PD becomes less independent and their quality of life decreases [8]. In addition, there may be visible slowing down of movement (bradykinesia), reduced amplitude of movement (hypokinesia), and/or difficulty initiating voluntary movements (akinesia) [12–14]. Symptoms of this disease are characteristic gait disturbances, visible as small, shuffling steps, and the lack of balance [15, 16]. These dysfunctions also concern motor coordination related to the work of the cerebellum, basal ganglia, and frontal lobes [17–19]. The lowest-level gait disorders result from damage to peripheral locomotor organs and sensory systems [20–23]. Middle-level gait disorders are related to the incorrect integration of sensory stimuli into the spatial map of movement and improper modulation of muscle strength in the structures of the subcortical nuclei, cerebellum, and cortical-spinal tracts [24, 25]. Damage in these areas causes hemiplegic and cerebellar gait and results from damage or dysfunction of the extrapyramidal system [26, 27]. High-level gait disorders include so-called cautious gait, subcortical balance, frontal balance, and isolated gait initiation (freezing) disorders, as well as frontal and psychogenic gait disturbances [28, 29]. The consequence of gait disturbances is the frequent loss of balance and falls. A fall, in its aftermath, can lead to many complications in the form of prolonged immobilisation, serious injury to the body, and, later, limitation of movement due to the fear of falling [30–33]. This causes a vicious cycle and even greater risk of falling [34]. Faced with difficulties in assessing the causes of falls, the aim of the study was to assess risk factors for falls in people with Parkinson's disease. The relationship between the risk of falling and the following factors was evaluated: subjective assessment of movement ability, the number of falls, the most troublesome symptoms of the disease, comorbidities, use of walking aids, disease stage and duration, physiotherapy, degree of fitness, type of injuries suffered during falls, other external factors, fear of falling, and the number of falls vs. the fear of falling.

## 2. Materials and Methods

The study comprised 53 participants, including 28 (52.8%) women and 25 (47.2%) men from the Association for Helping Patients with Parkinson's Disease in Kielce (Poland). The inclusion criteria were as follows: diagnosis of PD established according to the UK PD Society Brain Bank criteria at least 12 months prior to inclusion in the study, Hoehn and Yahr stages I-V, at least 1-year disease duration, and good response to levodopa therapy. The exclusion criteria were as follows: severe gait disability which made the person unable to walk unassisted, disorders other than PD which could have caused weakness or instability (stroke or myocardial infarction less than 3 months prior to the inclusion, severe dementia, severe hepatic or renal insufficiency, ankylosing spondylitis), a history of orthopaedic surgery of either hip or knee, other chronic disorders of the osteoarticular system, and labyrinthine disorders.

The research was performed at the beginning of 2020. Written consent was obtained from each participant for inclusion in the study. All participants were informed about its purpose and the possibility of withdrawing from participation at any stage. The patients were diagnosed by a neurologist in relation to PD. The study was conducted in accordance with the Declaration of Helsinki, and the protocol was approved by the Ethics Committee UJK No. 49/2020. At the beginning of the study, an original questionnaire created by the authors was conducted. This survey contained 20 questions. The first part included personal identification questions such as gender, age, weight, height, and place of residence. The detailed part included questions regarding disease course, accompanying symptoms, falls and their complications, external or internal factors that could increase the risk of falls, and physiotherapy. The asked questions regarded, among others, the frequency and type of physiotherapy. The Hoehn and Yahr 5-point disability scale was used to assess severity of Parkinson's disease [35]. The first (I) stage is the initial phase of the disease in which mobility impairment may be virtually absent. The second (II) stage consists of bilateral or axial symptoms. At the third (III) stage, imbalances begin to appear. A person at this stage is still able to lead an independent lifestyle and can work. Stage four (IV) is the full development of the disease. Moving around is much more difficult. At the fifth (V) stage, the ill person is completely dependent on other people. The Tinetti Balance and Gait Scale were used to assess the risk of falls [36]. The subjects were tested while being seated, standing up, standing, walking, and sitting down. Points were awarded for each instructed activity on a 0-2 point scale. The presence of walking aids was taken into account. Balance was assessed after performing 9 activities during which the maximum score was 16. Gait was assessed regarding 7 subpoints, for which the subject could obtain 12 points. A test score ≤ 18 points means a high risk of falling, a score between 19 and 23 points is a moderate risk, and a score ≥ 24 points is a low risk of falling. The Katz scale was used to assess the independence of people with PD [37]. This scale was implemented to evaluate performing basic activities of everyday living, such as bathing, dressing, undressing, using the toilet, moving around, eating, and controlled urine and stool excretion. The sum of the obtained points made it possible to assign a subject to one of the following groups: independent (5-6 points), moderately dependent (3-4 points), and significantly dependent (≤2 points). The Falls Efficacy Scale-International Short Form (FES-I) was used to assess evaluation of falling [38] during selected daily activities such as dressing or undressing, taking a bath or shower, sitting or getting up from a chair, climbing or descending stairs, reaching for something overhead or on the floor, climbing or descending an inclined surface, and going out to a social event. For each of these activities, the subjects could assign a value from 1 to 4, where 1 means that the person is not afraid of falling at all, while 4 means that he/she is very afraid. The sum of all points made it possible to assign the result to one of the three groups: 7-8—low fear of falling, 9-13—moderate fear of falling, and 14-28—high fear of falling.

### 2.1. Applied Statistical Methods

Basic statistical calculations such as mean, median, standard deviation, minimum, and maximum values were used in the study. For each variable, nonparametric tests were applied. To determine the relationship between variables, the chi-square test of independence was performed and the strength of the relationship between the two variables was measured using Cramér's *V* coefficient. A significance level of *p* < 0.05 was assumed.

## 3. Results

The participants considered imbalance the most troublesome symptom making them difficult to move around freely. Fewer patients indicated tremors, followed by stiffness and muscle weakness. The least number of people indicated small steps, difficulty in initiating movement, or slowing down as the most problematic. Only a few participants had no comorbidities. More than half also suffered from cardiovascular disease. Diabetes and urinary tract or metabolic diseases were also common. The smallest number of participants experienced rheumatological or respiratory diseases and depression. Parkinson's disease was most often diagnosed among the women and men aged 50-59 (39.6%). The largest group comprised subjects with a 10 to 19-year (49.1%) disease duration. Among the women under study, the most frequent duration of illness was 5-9 years (22.6%), while for men, this value totaled 1-4 years (15.1%). Cooccurring diseases included cardiovascular system diseases (31 patients) (58.49%), diabetes (15 patients) (28.3%), metabolic diseases (12 patients) (22.64%), depression (4 patients) (7.75%), respiratory system diseases (3 patients) (5.56%), digestive system diseases (7 patients) (13.21%), urinary tract diseases (15 patients) (28.30%), degenerative diseases (5 patients) (9.43%), and rheumatic diseases (2 patients) (3.77%). No other diseases were present in 7 (13.21%) of the patients. The most common phase of the disease according to the Hoehn and Yahr scale was stage III, equaling 16 subjects (30.2%).

### 3.1. Risk Factors for Falls

There was a significant relationship between the subjective assessment of the ability to move and the risk of falling (*χ*^2^ = 31.86, *p* < 0.001). People who rated their mobility as good or very good were at a low risk of falls. On the other hand, people with a high risk of falling assessed their walking as moderate, bad, or very bad. There was also a significant relationship between the number of falls and the risk of falls (*χ*^2^ = 37.92, *p* < 0.001). Most people who had never fallen were at a low risk of experiencing falls. In contrast, people who had fallen 4 times or more were at a high risk of falling (Table 1).

A significant relationship was observed between the subjects' independence and the risk of falls (*χ*^2^ = 19.28, *p* < 0.001). All independent people were at a low risk of falls. Those with a moderate risk of falls were also mostly self-sufficient. One person was moderately independent. Only in the group of people at a high risk of falls were the subjects significantly dependent. A significant relationship occurred between the type of injury suffered during a fall and the risk of falls (*χ*^2^ = 36.93, *p* < 0.001) (Table 2).

Among subjects at a high risk of falling, apart from bruising, there were also fractures, wounds, and cuts to the skin. Sprains concerned the smallest number of people from this group. In the last year, 9 (17.0%) people have fallen, 4 (7.55%) have fallen twice, 3 and 4 (7.55%) people have fallen 3 times, and 20 (37.7%) have fallen 4 or more times, while 16 (30.2%) people have never experienced a fall. A significant relationship was noted, among others, between external factors during the fall and the risk of falls (*χ*^2^ = 33.36, *p* < 0.001) (Table 3). Falls experienced by patients at high and moderate risk were mostly associated with another external factor. The most common were uneven surfaces or obstacles in the participants' way. A significant correlation could be noted between the number of falls among patients and the stage of the disease (*χ*^2^ = 45.34, *p* < 0.001). The majority of people with stage I of the disease had not fallen at all. Those with stage II of the disease usually did not fall or had fallen only once. In contrast, people with the disease at stage III had fallen 4 or more times. Those with the IV and V stages of the disease had also experienced falls 4 or more times (Table 3).

There was a significant relationship between fear of falling and the risk of falls (*χ*^2^ = 18.88, *p* < 0.001). People with low fall anxiety were mostly at a low risk of falls, while people with moderate to high fall anxiety were at high risk of falling. A significant relationship was found between the number of falls and fear of falling (*χ*^2^ = 33.49, *p* < 0.001). The majority of participants with low fall anxiety had never fallen. On the other hand, those with moderate and high anxiety of falling had most often experienced a fall 4 or more times (Table 4). There was no correlation between the applied physical therapy and the risk of falls (*χ*^2^ = 3.18, *p* = 0.17) (Table 4).

## 4. Discussion

Falls in PD often have serious consequences that require intensive care and prolonged immobilisation. Thromboembolic complications may also occur after a fall. On the other hand, there is a fear of subsequent falls, which clearly limits the patient's activity and causes further consequences of immobilisation [39]. Research on the causes and risks of falls in PD has been carried out for many years. The specific cause of a particular fall is usually difficult to determine because these patients often have multiple disorders. The causes of falls are generally divided into internal and external. The specific causes of falls in PD include postural instability, episodes of freezing and festination, Parkinsonian gait (or festinating gait), intensified dyskinesia, sudden falls, autonomic system disorders (orthostatic hypotension, neurocardiogenic syncope, and postural orthostatic tachycardia syndrome), neurological and sensory disturbances (lower limb muscle weakness, deep sensibility, cognitive, visual or balance impairment, and epileptic seizure), cardiovascular diseases, drugs, and environmental factors [40].

Faced with difficulties in assessing the causes of falls, many researchers have focused on evaluating the risk factors of falls [41]. In one study, the most common risk factors of falls were older age, longer duration of the disease, greater severity of symptoms, and freezing and trotting. The risk factors for falls also included a worse Timed Up and Go test result, the presence of posture disorders, and depression [39]. The presence of falls in the early stage of PD is a clear indication of nonidiopathic aetiology, such as progressive supranuclear palsy or vascular changes. In the study described above, the most common consequences of falls were skin damage and fractures to the wrist, the neck of the femoral bone, and transverse lesions. However, the most common causes of falls were environmental factors and so-called emergencies [41]. In this study, the most common injury was contusion, followed by fractures, while the least number of people had experienced superficial or sprained joints. Other authors pointed out that risk factors for falls were their prior occurrences, disease severity and duration, motor impairment, treatment with dopamine agonists, increased levodopa dosing, cognitive impairment, fear of falling, freezing gait, and decreased physical activity [42].

In this study, more than half of participants were at a high risk of falling. Most people rated their gait and ability to move as average or bad. People who fell at least once after being diagnosed with the disease were at a high risk of falls. The majority of individuals have already fallen 4 or more times. Most of them had sustained some sort of bodily injury, while 4 had to be immobilised as a result. External factors causing the falls were most often uneven surfaces and obstacles on the patient's path. Only 1 person identified slippery surfaces as a factor influencing the fall. The following are among internal factors that may potentially cause an increase in the risk of falls: taking more than 5 drugs, high blood pressure, visual disturbances, dizziness, diabetes, and hearing impairment.

Almost half of the respondents used aids while walking, most often in the form of one crutch, which could also increase the risk of falling. In another study, there have been correlations between attention deficit disorder and an increased risk of falling, possibly due to difficulties in performing compensatory movements [43]. No factors indicating a relationship between gender and age of the respondents and the risk of falling were found. In this study, the majority were women, and they were the ones who fell slightly more frequently. Nonetheless, the difference was not statistically significant. On the other hand, an increased risk of falls could be observed in those with a history of previous falls, with greater disease severity and its longer duration. The greatest number of participants were in stage III of the disease, and the least in stage V. However, there was no person with a low risk of falls above stage II. Along with an increase in the severity of the disease, the number of people who had never fallen before decreased. Nevertheless, a statistically significant relationship was not demonstrated between longer disease duration and a higher risk of falls.

Increased fear of falling can also contribute to the emergence of repeated falls. Anxiety can limit one's activity and, consequently, increase the risk of falls [44]. This is confirmed by the results of this study, in which majority of participants had a high fear of falling. Greater fear of falling was associated with a greater risk of falls. People with low fall anxiety were at a low risk of falling [45]. Most participants with a low risk of falling also had a low level of fear related to falling. Furthermore, most people with low anxiety levels had never fallen, while most people with high anxiety levels had fallen 4 times or more. Participants with moderate to high levels regarding fear of falling have mostly fallen within the last year. Therefore, in our opinion, people with PD should be covered with rehabilitation that reduces the level of anxiety [46].

Although many authors have demonstrated the significant role of physiotherapy in preventing falls among people with PD, such an observation was not noted in this trial. In our opinion, the reason for this was general selection of physical therapy exercises, limited to stretching and strengthening selected muscles and the lack of systematic implementation of them. Physiotherapy should be an integral part of PD treatment. It should play a very important role in the process of improving and adapting to living with the disease [47]. It should have significant impact on the symptoms occurring in the course of the disease but also on the general state of health. PD is different for each patient; thus, it is important to select individual therapy depending on motor and nonmotor symptoms, as well as general health of a patient [48]. Exercises recommended for patients with Parkinson's disease include breathing exercises, gait exercises, strengthening, balance, coordination, stretching, relaxation exercises, music therapy, dancing, and games improving motor ability [49]. Innovative techniques have been recently proposed such as virtual reality and exergaming, motor imagery and action observation, and robot-assisted physiotherapy [50]. Based on neuroplasticity, exercise increases synaptic strength and influences neurotransmission, thus potentiating functional circuitry in PD. Regular exercise, patient involvement, and implementation of the exercise programme, also at home, are prerequisites for the effectiveness of physiotherapy [50]. Greater emphasis should be placed here on exercises improving postural stability and balance, e.g., using the biofeedback method on stabilometric platforms [51]. Prevention is also a key factor in reducing falls. Patients with PD, as well as their families, are advised to arrange their homes in such a way that there are as few narrow, poorly lit corridors and stairs as possible or loose rugs that are easy to trip over by these patients. It is also worth installing handrails and handles, especially in the bathroom and toilet, where it is easy to lose postural stability and then fall [31].

## 5. Conclusion

Individuals who rated their mobility as good or excellent were at a low risk of falls. People who fell more times were at a high risk of falling. People who are more independent were at a low risk of falls. Previous injuries were the most associated with being at risk of falling. Uneven surfaces and obstacles on one's path are the external factors most associated with the risk of falling. People with low levels of fall anxiety were at a low risk of falls. Most people with low fall anxiety have never fallen. Additionally, the majority of patients with stage 1 of the disease have not fallen at all. The reason for the ineffectiveness of physiotherapy may be due to the exercise programs used and the lack of systematic implementation of them. PD is different for each patient; thus, it is important to select individually customized physiotherapy depending on motor and non-motor symptoms, as well as general health of a patient.

## Figures and Tables

**Table 1:** Subjective assessment of movement and the risk of falls and the number of falls versus the risk of falls.

Subjective assessment of movement and risk of falls											
Grading scale	Low		Moderate		High		Total		*χ* ^2^	*p*	*V*
	*N*	%	*N*	%	*N*	%	*N*	%			
Very good	3	5.66	0	0.00	0	0.00	3	5.7	31.86	<0.001	0.55
Good	5	9.43	1	1.89	2	3.77	8	15.1			
Moderate	3	5.66	11	20.75	12	22.6	26	49.1			
Bad	0	0.00	1	1.89	8	15.1	9	17.0			
Very bad	0	0.00	1	1.89	6	11.3	7	13.2			
Total	11	20.8	14	26.42	28	52.8	53	100.0			
Number of falls versus risk of falls											
Number of falls	Low		Moderate		High		Total		*χ* ^2^	*p*	*V*
	*N*	%	*N*	%	*N*	%	*N*	%			
1	1	1.89	5	9.43	3	5.77	9	17.0	37.92	<0.001	0.60
2	0	0.00	1	1.89	3	5.77	4	7.55			
3	0	0.00	0	0.00	4	7.69	4	7.55			
≥4	0	0.00	3	5.66	17	32.7	20	37.7			
No falls	10	18.9	5	9.43	1	1.92	16	30.2			
Total	11	20.8	14	26.42	28	53.8	53	100.0			

**Table 2:** Independence and the risk of falls and types of injuries suffered after a fall and the risk of falling.

Independence and risk of falls											
Level of independence	Low		Moderate		High		Total		*χ* ^2^	*p*	*V*
	*N*	%	*N*	%	*N*	%	*N*	%			
Independent	11	20.75	13	24.53	11	20.75	35	66.04	19.28	≤0.001	0.43
Moderately dependent	0	0.00	1	1.89	10	18.87	11	20.75			
Significantly dependent	0	0.00	0	0.00	7	13.21	7	13.21			
Total	11	20.75	14	26.42	28	52.83	53	100.0			
Types of injuries suffered following falls and risk of falling											
Type of suffered injury	Low		Moderate		High		Total		*χ* ^2^	*p*	*V*
	*N*	%	*N*	%	*N*	%	*N*	%			
Fractures	0	0.00	1	1.89	6	11.32	7	13.21	36.93	≤0.001	0.59
Contusion	0	0.00	2	3.77	10	18.87	12	22.64			
Wounds, cuts to the skin	0	0.00	1	1.89	4	7.55	5	9.43			
Sprains	1	1.89	0	0.00	1	1.89	2	3.77			
No injuries	0	0.00	5	9.43	11	20.75	16	30.19			
No falls	10	18.87	5	9.43	1	1.89	16	30.19			
Total	11	20.75	14	26.42	28	52.83	53	100.0			

**Table 3:** Other external factors during falls and the risk of falls and the number of falls and the stage of disease.

Other external factors during falls and the risk of falls															
Other external factors	Low				Moderate		High				Total		*χ* ^2^	*p*	*V*
	*N*		%		*N*	%	*N*		%		*N*	%			
Uneven surfaces	0		0.00		2	3.77	5		9.43		7	13.21	33.36	<0.001	0.65
Slippery surfaces	1		1.89		0	0.00	1		1.89		2	3.77			
Obstacles on path	0		0.00		1	1.89	5		9.43		6	11.32			
No external factors	0		0.00		6	11.32	16		30.19		22	41.51			
No falls	10		18.87		5	9.43	1		1.89		16	30.19			
Total	11		20.75		14	26.42	28		52.83		53	100.0			
Number of falls and disease stage															
Number of falls	I		II		III		IV		V		Total		*χ* ^2^	*p*	*V*
	*N*	%	*N*	%	*N*	%	*N*	%	*N*	%	*N*	%			
1	2	3.77	2	3.77	4	7.55	1	1.89	0	0.00	9	16.98	45.34	<0.001	0.46
2	0	0.00	1	1.89	2	3.77	1	1.89	0	0.00	4	7.55			
3	0	0.00	0	0.00	1	1.89	2	3.77	1	1.89	4	7.55			
≥4	0	0.00	0	0.00	8	15.09	10	18.87	2	3.77	20	37.74			
Never	11	20.75	4	7.55	1	1.89	0	0.00	0	0.00	16	30.19			
Total	13	24.53	7	13.21	16	30.19	14	26.42	3	5.66	53	100.0			

**Table 4:** Fear of falling and risk of falls, number of falls and fear of falling, and physiotherapy and risk of falls.

Fear of falling and risk of falls											
Anxiety level	Low		Moderate		High		Total		*χ* ^2^	*p*	*V*
	*N*	%	*N*	%	*N*	%	*N*	%			
Low	8	15.09	1	1.89	2	3.77	11	20.75	18.88	≤0.001	0.42
Moderate	3	5.66	5	9.43	6	11.32	14	26.42			
Very high	2	3.77	9	16.98	17	32.08	28	52.83			
Total	13	24.53	15	28.30	25	47.17	53	100.0			
Number of falls and fear of falling											
Number of falls	Low		Moderate		High		Total		*χ* ^2^	*p*	*V*
	*N*	%	*N*	%	*N*	%	*N*	%			
1	1	1.89	4	7.55	4	7.55	9	16.98	33.49	≤0.001	0.56
2	0	0.00	1	1.89	3	5.66	4	7.55			
3	0	0.00	1	1.89	3	5.66	4	7.55			
≥4	0	0.00	7	13.21	13	24.53	20	37.74			
Never	12	22.64	2	3.77	2	3.77	16	30.19			
Total	13	24.53	15	28.30	25	47.17	53	100.0			
Physiotherapy and risk of falls											
	Current		In the past year		Never applied		Total		*χ* ^2^	*p*	*V*
	*N*	%	*N*	%	*N*	%	*N*	%			
	7	13.21	3	5.66	1	1.89	11	20.75			
	11	20.75	2	3.77	1	1.89	14	26.42	3.18	0.17	0.53
	22	41.51	2	3.77	4	7.55	28	52.83			
	40	75.47	7	13.21	6	11.32	53	100.0			

## Data Availability

The data used to support the findings of this study are available from the corresponding author upon request.
